# P-2047. Public Health Impact and Economic Value of an Additional Dose of Pfizer-BioNTech XBB.1.5-adapted COVID-19 Vaccine for Older Adults in the United States

**DOI:** 10.1093/ofid/ofae631.2203

**Published:** 2025-01-29

**Authors:** Alon Yehoshua, Benjamin Yarnoff, Manuela Di Fusco, Santiago M C Lopez, Elizabeth A Thoburn, Abby Rudolph, Kinga Marczell

**Affiliations:** Pfizer Inc., New York, New York; Evidera Inc, Bethesda, Maryland; Pfizer Inc, New York, New York; Pfizer Inc, New York, New York; Pfizer, Inc, Collegeville, PA; Pfizer Inc, New York, New York; Evidera Ltd, Budapest, Pest, Hungary

## Abstract

**Background:**

To assess the public health impact and economic value of an additional dose of BNT162b2 XBB.1.5-adapted COVID-19 vaccine 6 months after initial dose for those aged ≥65 years, compared to a single dose in the United States.

**Table 1.**
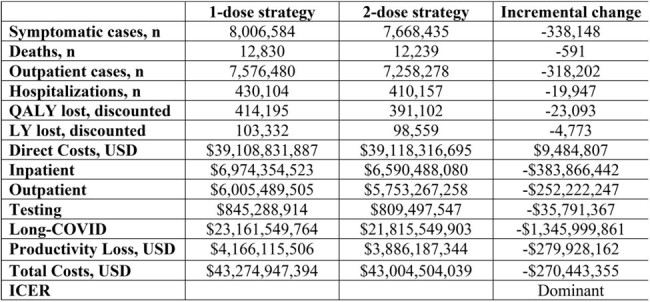
Clinical and Economic Outcomes of 2-dose vs 1-dose Strategy in the Population Aged ≥65 Years. LY = life year; QALY = quality adjusted life year; ICER = incremental cost effectiveness ratio

**Methods:**

A combined Markov and decision tree model was used to estimate the public health impact of those aged ≥65 years receiving an additional BNT162b2 XBB.1.5-adapted dose over for a one-year time horizon (Oct 2023-Sept 2024). Age-stratified (65-74, 75+) annual attack, hospitalization, and vaccine coverage rates were informed by public health national surveillance data. Other age-stratified clinical, cost, and vaccine effectiveness parameters were informed by publications. Parameter uncertainty was examined through scenario and sensitivity analyses. Conservatively, indirect effects (e.g. reduced transmission) and broad societal benefits were not considered.

**Results:**

For individuals aged 65 years and older, the model demonstrated that compared to a single dose, an additional dose of the BNT162b2 XBB.1.5-adapted COVID-19 Vaccine resulted in 338,148 fewer symptomatic cases, 19,947 fewer hospitalizations, and 591 fewer deaths. Cost of additional vaccinations were offset by costs averted (e.g., Long COVID (∼$1.3B), inpatient (∼$384M), indirect (∼$280M), and outpatient (∼$252M) costs), leading to a total cost savings of ∼$270.5M. The two-dose strategy for older adults improved health outcomes and lowered costs resulting in a dominant ICER (Table 1). The additional dose was predicted to be a budget-efficient solution for payers. Results were most sensitive to variation in the symptomatic rate of infection and the hospitalization rate.

**Conclusion:**

An additional dose of BNT162b2 XBB.1.5-adapted COVID-19 vaccination for adults aged 65 years and older in the US may generate notable reductions in symptomatic cases, hospitalizations, and deaths and result in cost savings. The results demonstrate that administering an additional COVID-19 vaccine dose to older adults in the US should be an important public health goal, supporting current ACIP and CDC recommendations.

**Disclosures:**

Alon Yehoshua, PharmD, MS, Pfizer Inc: Employer|Pfizer Inc: Stocks/Bonds (Public Company) Benjamin Yarnoff, PhD, Pfizer Inc: Grant/Research Support Manuela Di Fusco, PhD, Pfizer Inc.: Employee|Pfizer Inc.: Stocks/Bonds (Public Company) Santiago M.C. Lopez, MD, Pfizer Inc.: Employee|Pfizer Inc.: Stocks/Bonds (Public Company) Elizabeth A. Thoburn, MPH, Pfizer Inc: Employer|Pfizer Inc: Stocks/Bonds (Public Company) Abby Rudolph, PhD, Pfizer Inc: Employer|Pfizer Inc: Stocks/Bonds (Public Company) Kinga Marczell, PhD, Pfizer Inc: Grant/Research Support

